# Supplementary material for: VolcaNoseR is a web app for creating, exploring, labeling and sharing volcano plots
Source: Sci Rep. 2020 Nov 25;10:20560. doi: 10.1038/s41598-020-76603-3 (PMC7689420; doi:10.1038/s41598-020-76603-3)
Supplement: Supplementary file 1 — Supplementary Information [file 41598_2020_76603_MOESM1_ESM.docx]

VolcaNoseR is a web app for creating, exploring, labeling and sharing volcano plots

Joachim Goedhart^1, ‡^ and Martijn S. Luijsterburg^2^

^1^Swammerdam Institute for Life Sciences, Section of Molecular Cytology, van Leeuwenhoek Centre for Advanced Microscopy, University of Amsterdam, P.O. Box 94215, NL-1090 GE Amsterdam, The Netherlands.

Email: [j.goedhart@uva.nl](mailto:j.goedhart@uva.nl) | Twitter: [@joachimgoedhart](https://twitter.com/joachimgoedhart) | ORCID: [0000-0002-0630-3825](https://orcid.org/0000-0002-0630-3825)

^2^Department of Human Genetics, Leiden University Medical Center, Einthovenweg 20, 2333 ZC, Leiden, The Netherlands.

Email: [M.Luijsterburg@lumc.nl](mailto:M.Luijsterburg@lumc.nl) | Twitter: [@luijsterburglab](https://twitter.com/@luijsterburglab) | ORCID: [0000-0001-5796-6541](https://orcid.org/0000-0001-5796-6541)

**S1 Text – Passing parameters to VolcaNoseR through the HTML address**

Passing parameters through the HTML address can be used to define a visualization and alter the standard layout. There are several queries (?data, ?vis, ..) that can be used. Each of the queries can hold multiple parameters. The queries are separated by an ampersand (&) and the parameters are separated by a semicolon(;).

Parameters that need no change (i.e. remain default) should be left empty. The parameters are defined by their position so their order is critical. Below, the parameters that can be changed through the HTML address are indicated, with the options between brackets:

?data

[1] data_input {1/2/3/4/5}

[2] tidyInput {T}

[3] x_var variable (text)

[4] y_var variable (text)

[5] g_var variable (text)

?vis

[1] pointSize {0-10}

[2] alphaInput {0-1}

[3] fc_cutoff Numeric input from double slider, e.g.: -2,2

[4] p_cutoff Numeric input from slider, e.g.: 2

[5] direction {all | significant | increased | decreased}

[6] criterion {manh | euclid | fc | sig}

?can

[1] top_x Numeric

[2] show_table {T | F}

[3] hide_labels {T | F}

[4] user_gene_list list of comma-separated names

?layout

[1] rotate_plot {T | F}

[2] add_grid {T | F}

[3] change_scale {T | F}

[4] range_x Numeric (minimum value, maximum value)

[5] range_y Numeric (minimum value, maximum value)

[6] For compatibility ‘X’

[7] plot_height Numeric (pixels)

[8] plot_width Numeric (pixels)

?color

[1] adjustcolors {1 | 3 | 5}

[2] user_color_list list of colors separated by commas

?label

[1] add_title {T | F}

[2] title Text

[3] label_axes {T | F}

[4] lab_x Text

[5] lab_y Text

[6] adj_fnt_sz {T | F}

[7] fnt_sz_title Numeric

[8] fnt_sz_labs Numeric

[9] fnt_sz_ax Numeric

[10] fnt_sz_cand Numeric

[11] add_legend {T | F}

?url

query[[‘url’]] URL URL
